# Diagnostic Performance of CT-like Images for Lumbar Pedicle Screw Planning and Spinal Canal Area Measurement: A Comparative Study with Conventional CT and MRI

**DOI:** 10.3390/tomography12030040

**Published:** 2026-03-16

**Authors:** Akira Ogihara, Takeshi Fukuda, Shunsuke Katsumi, Hiroya Ojiri

**Affiliations:** 1 Department of Radiology, The Jikei University School of Medicine, Minato-ku, Tokyo 105-8461, Japan; a.ogihara.fw@jikei.ac.jp (A.O.);; 2 Department of Orthopedic Surgery, The Jikei University School of Medicine, Minato-ku, Tokyo 105-8461, Japan

**Keywords:** CT-like images, pedicle screw, CT, MRI

## Abstract

**Background**: Although magnetic resonance imaging (MRI) provides excellent soft-tissue contrast for most spinal evaluations, computed tomography (CT) is still always required for preoperative planning to assess osseous anatomy and determine surgical device size, increasing the radiation exposure and workflow complexity. CT-like images enable visualization of precise bone morphology without ionizing radiation. In addition, these images often provide CT myelography-like contrasts, allowing the simultaneous depiction of the spinal canal area (SCA). This study aimed to evaluate whether CT-like images provide measurement accuracy equivalent to conventional CT and MRI for pedicle screw planning and spinal canal area assessment. **Methods**: We retrospectively analyzed paired lumbar CT and MRI datasets obtained within ≤1 month in 51 patients. Pedicle width and length were measured on CT and CT-like images, whereas SCA was measured on T2 weighed-images and CT-like images. A total of 224 vertebrae were analyzed. Annotated images were independently evaluated by two readers in a randomized order. Inter-modality agreement was assessed using intraclass correlation coefficients (ICCs) and a Bland–Altman analysis. **Results**: CT-like images demonstrated an excellent agreement with CT for pedicle measurements (ICCs: 0.968–0.985 for width; 0.922–0.966 for length). Mean differences were ≤0.1 mm for pedicle width and approximately 1 mm for pedicle length, which are unlikely to affect screw selection. The agreement with T2WI for SCA was good to excellent (ICCs: 0.766–0.945). **Conclusions**: CT-like images provide comparable performance for quantitative pedicle assessment and show high agreement for SCA evaluation, supporting comprehensive preoperative assessment with a single MRI examination.

## Simple Summary

Computed tomography (CT) is often required before spine surgery to measure bone anatomy, even though magnetic resonance imaging (MRI) is already performed for nerve and soft-tissue evaluation. This leads to additional radiation exposure and increased clinical workloads. In this study, we evaluated whether CT-like images generated from MRI can provide measurements comparable to conventional CT for pedicle screw selection and assessing the spinal canal. Our results show that CT-like images offer reliable and clinically acceptable measurements. These findings suggest that a single MRI examination may support comprehensive preoperative spinal assessment, potentially reduce radiation exposure and improve clinical workflow.

## 1. Introduction

MRI is routinely performed for spinal imaging because of its superior ability to depict neural and soft-tissue structures. However, for preoperative evaluation, surgical navigation, and instrumentation planning, CT is often additionally acquired owing to its excellent visualization of osseous anatomy [1]. This additional CT examination increases radiation exposure, healthcare costs, and workflow complexity.

Recently, several MRI techniques designed for bone visualization—such as black-bone imaging, ultrashort echo time (UTE), zero echo time (ZTE), and three-dimensional T1-weighted gradient-echo (3D T1GRE) sequences—have been developed to generate CT-like images that allow assessment of bone morphology without ionizing radiation within a single examination [2,3]. Interest in CT-like images have rapidly increased in recent years, and multiple studies have reported that MRI-derived CT-like images provide diagnostic performance comparable to conventional CT for depicting cortical bone and osseous anatomy [2,3,4,5].

In spinal fusion surgery, one of the most commonly performed spinal procedures, pedicle screws are widely used [1]. To prevent complications such as neural, vascular or organ injury, screw loosening, and vertebral fracture, detailed anatomical assessment during preoperative planning is essential. In particular, selecting an optimal screw size is critical. Increasing the pedicle screw diameter within the anatomical limits has been shown to significantly improve fatigue strength, pullout resistance, and overall construct stability, whereas the longest feasible screw length has been associated with enhanced resistance to lateral bending and axial rotation, as well as reduced mechanical stress at the bone–screw interface [6,7]. Key anatomical parameters that determine the optimal screw size include pedicle width and length [8,9,10]. Previous studies comparing conventional MRI and CT have consistently demonstrated that MRI-based pedicle measurements lack sufficient reliability for surgical planning [5,11]. Meanwhile, previous studies that assess CT-like images for pedicle screw selection remain limited in sample size, and no statistical analyses have yet been reported [4].

Moreover, CT-like images based on 3D T1GRE sequences with grayscale inversion often yields high signal intensity of cerebrospinal fluid, providing CT myelography-like contrast. Previous studies have shown that quantitative assessment of the SCA, defined as the cross-sectional area of the dural sac measured on T2-weighted images or CT myelography, correlates with symptom severity and functional impairment in patients with lumbar spinal stenosis [12,13,14,15]. These findings suggest that spinal canal stenosis may be assessed simultaneously using CT-like images without CT myelography. However, SCA measurements using CT-like images have not yet been systematically validated.

Therefore, this study aims to evaluate whether CT-like images provide measurement accuracy equivalent to that of conventional CT and MRI for assessing the pedicle morphology and spinal canal area. By establishing this equivalence, we sought to enable comprehensive preoperative spinal assessment within a single MRI examination, thereby improving workflow efficiency and reducing radiation exposure.

## 2. Materials and Methods

This study was approved by the institutional ethics committee of our hospital. Paired lumbar CT and MRI datasets were retrospectively collected from patients who underwent both examinations between December 2023 and July 2024. The interval between CT and MRI examinations was restricted to within one month. Patients were excluded if (1) postoperative changes or metallic implants were present in the target region or (2) the imaging planes of CT and MRI differed substantially, precluding reliable inter-modality comparison.

MRI examinations were performed using either a 3T Magnetom Lumina or Magnetom Skyra system (Siemens Healthineers, Erlangen, Germany). CT-like images were obtained using a 3D T1GRE-based sequence. Imaging parameters included an field of view (FOV) of 300 × 210 mm; TR of 12 ms; dual-echo acquisition with echo times of 2.46 ms and 7.38 ms followed by summation processing; and a 3D acquisition matrix of 320 × 304 × 304, corresponding to an isotropic voxel size of 0.9 mm. Additional parameters included a flip angle of 10°, one signal average, and a total acquisition time of 3 min 50 s. CT-like images were generated by inverting the grayscale of the original magnitude images, resulting in high signal intensity for tissues that are characteristically hypointense on original T1-weighted images, such as cortical bone.

Axial T2-weighted images (T2WI), acquired as part of the routine clinical protocol, were used for spinal canal area analysis. Imaging parameters for the fast spin-echo T2WI sequences were as follows: FOV, 180 × 144 mm; repetition time (TR)/echo time (TE), 4000/84 ms; in-plane matrix, 336 × 235; slice thickness, 3 mm; and acquisition time, 2 min 20 s.

CT examinations were performed using a 64–detector row CT scanner (SOMATOM Definition Edge; Siemens Healthineers, Erlangen, Germany). Scanning parameters were as follows: tube voltage of 100–120 kVp, automatically optimized using CARE kV; tube current of 155–306 mA with automatic tube current modulation (CARE Dose 4D); rotation time of 0.8 s; and collimation of 64 × 0.6 mm. Images were reconstructed with a slice thickness of 1.0 mm and a reconstruction interval of 0.8 mm.

Image analyses were independently performed by two board-certified radiologists, each with more than seven years of clinical experience. Pedicle width, pedicle length, and spinal canal area were all measured on CT-like images. For reference, pedicle width and length were assessed on CT, whereas spinal canal area was assessed on T2-weighted images. All measurements were performed on a commercially available workstation (VINCENT; Fujifilm Corporation, Tokyo, Japan). Image evaluation was randomized and performed in separate sessions, with observers blinded to patient-related information and without access to other imaging modalities during each assessment.

For each lumbar intervertebral level (from L1/2 to L5/S), axial images were reformatted to be parallel to the vertebral endplate. Automated 3D rigid registration between CT and MRI datasets was not performed. Because different reference sequences were used across modalities, slight discrepancies in slice level could occur; however, slices were carefully selected to correspond to the same anatomical level as closely as possible.

1. **Pedicle width and length**

**Figure 1:**
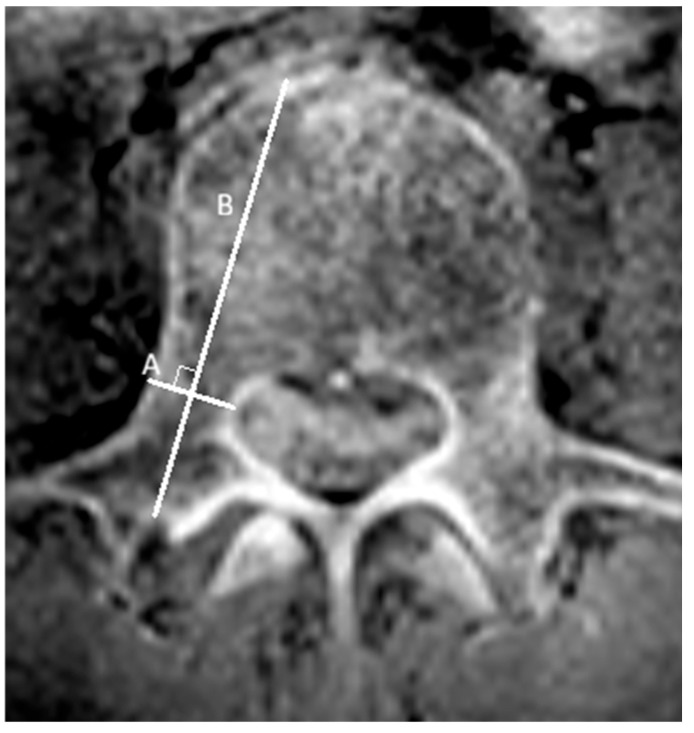
Measurement of pedicle dimensions. The pedicle width (A) is measured as the line representing the narrowest portion (isthmus) of the pedicle. The pedicle length (B) is measured along the perpendicular bisector of line A, extending from the anterior cortex of the vertebral body to the dorsal cortex of the transverse process.

On CT and CT-like images, pedicle width was defined as the distance between the outer cortical margins at the narrowest portion (isthmus) of the pedicle. Pedicle length was defined as the distance from the anterior vertebral cortical margin to the posterior cortical margin of the transverse process, measured along the perpendicular bisector of the width line (Figure 1).

2. **Spinal canal area**

**Figure 2:**
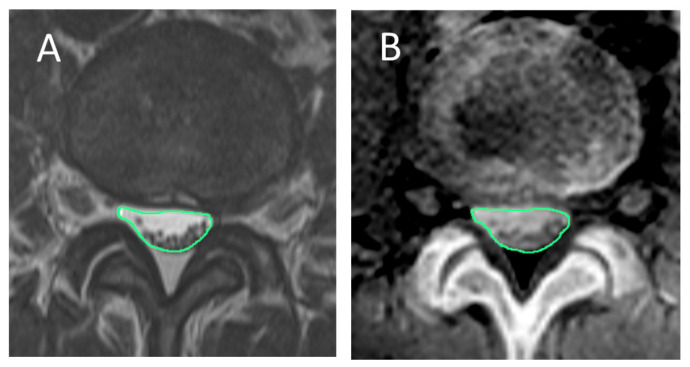
Measurement of spinal canal area. On the same axial cross-sections of both (**A**) T2-weighted image and (**B**) CT-like image, the spinal canal area was measured by manually tracing along the inner border of the dura mater.

On T2-weighted and CT-like images, a region of interest (ROI) was manually traced along the inner border of the dura mater, and the enclosed area was recorded as the spinal canal area (Figure 2).

The normality of continuous variables (pedicle width, pedicle length, and spinal canal area) was assessed using the Shapiro–Wilk test. As none of the variables significantly deviated from normality, parametric statistical methods were applied. Inter-modality agreement between CT and CT-like images were evaluated using intraclass correlation coefficients (ICCs) with a two-way random-effects model for absolute agreement. ICC values were interpreted as follows: poor (<0.50), moderate (0.50–0.75), good (0.75–0.90), and excellent (>0.90). Agreement between measurement methods was further assessed using Bland–Altman analysis to calculate the mean difference (bias) and 95% limits of agreement (LOAs). To evaluate whether measurement discrepancies differed across vertebral levels, the absolute differences between CT and CT-like measurements were calculated and compared among lumbar levels (L1–L5) using one-way analysis of variance (ANOVA) with Tukey post hoc testing. All statistical analyses were performed using SPSS software (version 28.0; IBM Corp., Armonk, NY, USA), and a *p*-value < 0.05 was considered statistically significant.

## 3. Results

**Figure 3:**
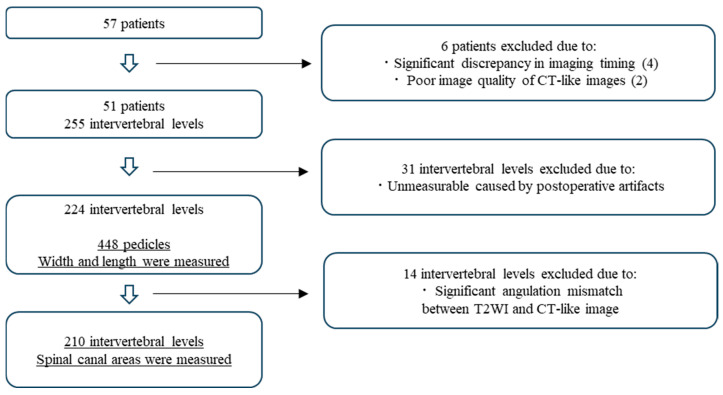
Patient flowchart demonstrating selection process.

A total of 51 patients were included, yielding paired CT and MRI datasets comprising 224 vertebrae, 448 paired pedicles, and 210 intervertebral spinal canal areas (Figure 3). The mean age of the patients was 63.1 ± 18.4 years (range, 14–92 years), and 24 patients (47.1%) were male. The average values of the measurements obtained by the two observers for pedicle width and pedicle length are summarized in Table 1. The absolute differences between CT and CT-like measurements for pedicle width and length are also summarized in Table 1. The largest mean absolute difference was observed at L5. One-way ANOVA demonstrated a significant difference among vertebral levels, and post hoc Tukey tests showed significantly larger discrepancies at L5 compared with L1 (*p* < 0.001) and L2 (*p* = 0.015). Similarly, the largest absolute difference for pedicle length was observed at L5, and post hoc Tukey tests demonstrated significantly greater differences at L5 compared with L1 (*p* = 0.001) and L4 (*p* = 0.010). Because the study population included patients with degenerative spinal conditions, absolute SCA values were expected to show substantial variability and were therefore not presented as descriptive statistics.

### 3.1. Intraclass Correlation Coefficient (ICC) Analysis

For observer 1, ICCs between CT and CT-like images were 0.985 (95% CI, 0.981–0.988) for pedicle width, 0.922 (95% CI, 0.904–0.936) for pedicle length, and 0.945 (95% CI, 0.927–0.959) for spinal canal area (all *p* < 0.001). For observer 2, ICCs were 0.968 (95% CI, 0.960–0.974) for pedicle width, 0.966 (95% CI, 0.954–0.975) for pedicle length, and 0.766 (95% CI, 0.719–0.806) for spinal canal area (all *p* < 0.001).

Both observers demonstrated excellent agreement between CT and CT-like images for pedicle width and length (ICC > 0.90). For spinal canal area measurements, agreement ranged from moderate to excellent.

### 3.2. Bland–Altman Analysiss

Bland–Altman analysis showed that, for both pedicle width and pedicle length, the majority of measurements fell within the 95% limits of agreement.

**Figure 4:**
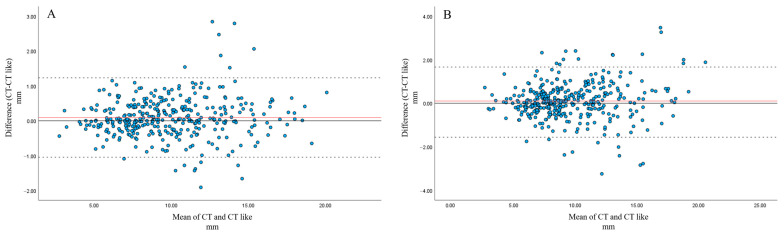
Bland–Altman analysis of pedicle width measurements between CT and CT-like images by observer 1 (**A**) and observer 2 (**B**). (**A**) Bland–Altman analysis for observer 1 demonstrated a minimal mean difference (0.09 mm), with narrow 95% limits of agreement (approximately −1.0 to +1.2 mm). (**B**) Bland–Altman analysis for observer 2 also demonstrated a minimal mean difference (0.10 mm), with narrow 95% limits of agreement (approximately −1.6 to +1.8 mm) for pedicle width.

For pedicle width, mean differences were 0.0915 mm and 0.1029 mm, with CT yielding slightly larger values than CT-like images for both observers (Figure 4).

**Figure 5:**
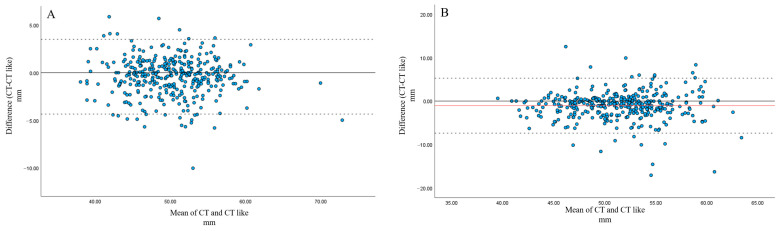
Bland–Altman analysis of pedicle length measurements between CT and CT-like images by observer 1 (**A**) and observer 2 (**B**). (**A**) Bland–Altman analysis for observer 1 demonstrated a small mean difference (0.42 mm), with acceptable 95% limits of agreement (approximately −4.4 to +3.5 mm) for pedicle length. (**B**) Bland–Altman analysis for observer 2 demonstrated a small mean difference (1.06 mm), with wider 95% limits of agreement (approximately −7.4 to +5.3 mm) for pedicle length.

For pedicle length, mean differences were 0.421 mm and 1.057 mm, with CT yielding slightly smaller values than CT-like images (Figure 5).

**Figure 6:**
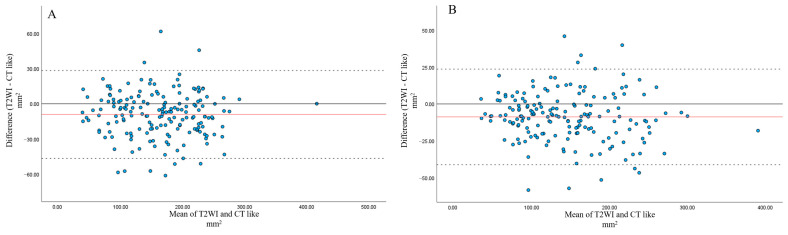
Bland–Altman analysis of spinal canal area measurements between CT-like images and T2WI by observer 1 (**A**) and observer 2 (**B**). (**A**) Bland–Altman analysis for observer 1 demonstrated a small mean difference (9.16 mm^2^), with acceptable 95% limits of agreement (approximately −46.6 to +28.3 mm^2^). (**B**) Bland–Altman analysis for observer 2 demonstrated a small mean difference (8.82 mm^2^), with 95% limits of agreement (approximately −41.2 to +23.6 mm^2^).

For the spinal canal area, slight variability was observed between T2-weighted MRI and CT-like images. However, most measurements were within the 95% LOA. Mean differences were 8.82 mm^2^ and 9.16 mm^2^, with T2-weighted MRI tending to yield smaller values than CT-like images for both observers (Figure 6).

## 4. Discussion

In this study, we demonstrated that CT-like images allow reliable quantitative assessment of the pedicle morphology and SCA, with measurement agreement comparable to conventional CT and MRI respectively. Pedicle width and length measurements showed consistently excellent agreement across modalities, whereas SCA measurements exhibited greater inter-modality variability but remained within acceptable LOAs. These findings indicate that CT-like images can provide robust morphometric information for preoperative spinal assessment.

Accurate preoperative selection of pedicle screws is essential to prevent complications such as neural, vascular or organ injury, screw loosening, and vertebral fracture [7,8,9,16,17]. Although larger screws generally improve fixation strength, excessively large screws increase the risk of cortical breach and anterior overpenetration. Previous studies have shown that safe pedicle screw insertion depth is determined as a proportion of vertebral body length, balancing fixation strength with the risk of anterior cortical violation [8]. In parallel, biomechanical and clinical studies support selecting a screw diameter occupying approximately 70–80% of the pedicle width while preserving a minimal cortical margin to reduce the risk of pedicle violation and maintain pullout strength [9,10]. Experimental studies have further demonstrated that screw length and diameter influence fixation stability under cyclic loading conditions, particularly in compromised bone quality [7,18], while inappropriate screw selection increases the risk of pedicle breach [19].

The pedicle width and length measurements obtained in this study were generally consistent with previously reported morphometric data of the lumbar spine [20]. In particular, the well-known caudal increase in pedicle width and the pattern of pedicle length peaking at L3–L4 with a slight decrease at L5 were observed not only on CT but also on CT-like images, supporting the anatomical validity of CT-like measurements.

In our cohort, pedicle length and width measurements obtained from CT-like images showed excellent agreement with those obtained from CT, with mean differences of <0.1 mm for width and <1 mm for length. Given that commercially available pedicle screws are typically manufactured in 0.5 mm increments for diameter and 5 mm increments for length, the observed inter-modality differences are unlikely to affect screw selection in most clinical situations [1,10]. Therefore, the observed agreement between CT and CT-like images is unlikely to translate into clinically meaningful differences during actual screw insertion. These findings support the feasibility of pedicle screw planning using a single MRI examination with measurement accuracy comparable to that of CT. Although the LOAs for pedicle length were larger than those for pedicle width, prior biomechanical studies have demonstrated that screw diameter is a primary determinant of fixation strength, whereas screw length plays a secondary role once sufficient purchase is achieved, supporting the clinical relevance of the observed agreement for pedicle width [21].

In the Bland–Altman analysis, occasional outliers with discrepancies exceeding 10 mm in pedicle length were observed. Measurements of longer anatomical structures appeared more susceptible to such discrepancies, particularly at the lower lumbar levels. In level-specific analysis, the largest mean absolute differences were observed at L5, supporting the notion that measurement variability tends to increase at levels with steeper lumbosacral angulation. This variability likely reflects the influence of lumbar lordosis and the steep lumbosacral angle, which make it technically challenging to achieve perfectly identical slice orientations between CT and MRI during manual slice matching. Even small differences in slice orientation may lead to relatively large discrepancies in linear measurements, particularly for pedicle length. Consequently, implementing standardized 3D reformatting and automated registration protocols in future studies may minimize these orientation-related errors and further improve inter-modality agreement. In addition to slice orientation mismatch, MRI-specific physical phenomena associated with CT-like image may also contribute to these discrepancies. These include chemical shift artifacts. Blooming effect, and partial volume effect, which may collectively influence the apparent boundaries of cortical bone and slightly affect linear measurement when compared with CT.

Lumbar spinal stenosis is characterized by narrowing of the spinal canal due to degenerative changes such as osteophyte formation, disc bulging or herniation, and hypertrophy of the ligamentum flavum, leading to low back pain and neurological symptoms. The severity of stenosis can be evaluated using both qualitative grading systems and quantitative metrics [12,13,14,15,22]. Among quantitative measures, SCA has been shown to correlate with symptom severity, walking capacity, pain, and quality of life in patients with lumbar spinal stenosis, supporting its role as a clinically meaningful reference parameter for central canal narrowing [12,13,14,15]. This makes SCA a reliable and clinically meaningful reference parameter for evaluating central canal stenosis severity.

In the present study, SCA measurements obtained from CT-like images demonstrated good to excellent agreement with T2WI. Although greater inter-modality variability was observed compared with pedicle measurements, these discrepancies remained well within clinically acceptable limits. T2WI tended to yield slightly smaller SCA values, likely due to its superior soft-tissue contrast for delineating the ligamentum flavum and intervertebral discs. However, this trend did not result in systematic overestimation that would mask the severity of spinal stenosis. Bland–Altman analysis confirmed that measurement differences were largely confined within the 95% limits of agreement, with only minimal mean differences. These findings indicate that CT-like images provide a robust and reliable assessment of the patent canal area and may be sufficient for routine preoperative spinal evaluation without compromising diagnostic accuracy.

The acquisition time for CT-like images is less than 4 min, which inevitably adds modestly to the total MRI scan time. However, this approach avoids radiation exposure and may streamline the diagnostic workflow, reduce patient burden, and offer advantages from a healthcare economic perspective by eliminating the need for an additional CT examination.

This study has several limitations. First, although the interval between CT and MRI examinations was restricted to within one month, subtle anatomical or positional changes between examinations, as well as minor discrepancies in slice orientation, may have introduced measurement variability. In this study, automated 3D rigid registration software was not utilized, and measurements relied on manual slice selection. As indicated by the Bland–Altman analysis, this approach may have contributed to increased variability at the lower lumbar levels, where the steep lumbosacral angle makes precise anatomical matching between different imaging tables more difficult. The implementation of standardized 3D reformatting and rigid registration protocols in future studies could potentially further reduce this source of variability and enhance the agreement rates. Second, acceptable measurement errors may vary depending on surgical technique and instrumentation specifications. Therefore, while CT-like images demonstrated measurement accuracy comparable to CT at the population level, their clinical applicability should be interpreted in the context of individual surgical requirements. Third, because this study was retrospective, the imaging measurements were not correlated with intraoperative surgical outcomes or pedicle screw placement success rates. Nevertheless, because conventional CT remains the current gold standard for preoperative pedicle screw planning, we believe that a head-to-head comparison between CT and CT-like images holds substantial clinical significance. Forth, the sample size of the present study remains limited, and the analysis was confined to the lumbar spine in a single-center setting. Further investigations with larger cohorts, including the cervical and thoracic spine, are warranted to confirm the generalizability and reproducibility of these findings across different spinal levels and institutions. 

## 5. Conclusions

In conclusion, CT-like images provide measurement accuracy comparable to conventional CT for quantitative assessment of pedicle morphology and demonstrate good agreement with MRI for SCA evaluation. These findings indicate that CT-like images can support comprehensive preoperative spinal assessment using a single MRI examination, with the potential to reduce radiation exposure and streamline clinical workflows.

## Figures and Tables

**Table 1:** Level-specific measurements of pedicle width and pedicle length.

	Pedicle Width			Pedicle Length		
	CT-like Image	CT	Absolute Differences	CT-like Image	CT	Absolute Differences
L1	6.841 ± 1.785	6.958 ± 1.854	0.348 ± 0.295	50.725 ± 4.395	49.945 ± 4.066	1.481 ± 1.663
L2	7.366 ± 1.457	7.399 ± 1.521	0.477 ± 0.747	51.438 ± 4.480	50.906 ± 4.568	2.047 ± 4.279
L3	9.209 ± 1.982	9.18 ± 1.871	0.579 ± 0.897	51.581 ± 4.583	51.64 ± 4.423	1.903 ± 1.978
L4	10.834 ± 1.888	10.878 ± 1.782	0.550 ± 0.546	50.241 ± 5.241	51.08 ± 5.452	1.682 ± 2.060
L5	14.324 ± 2.426	14.679 ± 2.386	0.738 ± 0.752	48.969 ± 5.212	50.398 ± 5.767	2.725 ± 2.646

Values are presented as mean ± standard deviation.

## Data Availability

The data presented in this study are not publicly available due to ethical and privacy restrictions but are available from the corresponding author upon reasonable request.

## Abbreviations

The following abbreviations are used in this manuscript:

SCA	Spinal canal area
ROI	Region of interest
LOA	Limit of agreement
